# Selections

**Published:** 1893-11

**Authors:** 


### Selections.

### A SIMPLE AND EFFICIENT STRENGTHENER FOR LOWER VULCANITE DENTURES.

Mr. Walter Coffin described a simple and easily made strengthener for inserting in vulcanite, especially adapted for inferior dentures, partial or whole, which he had used extensively with great satisfaction for many years. It consisted of a metallic strip or wire of any section, preferably of oval or half-round platinum wire, and then wound or wrapped from end to end in an open spiral with a thin gold wire about the size of which the ordinary gold springs are made, the whole soldered together with very small pieces or filings of gold solder. The platinum-wire is first bent as accurately as possible to fit the model, then wrapped and soldered. It then becomes very rigid, but still slightly elastic in all directions. Any clasps, bands, or gold backings being used may be soldered to the strengthener. When not so held in place, a length of the thin wrapping-wire may be left free at both ends of the strengthener and caught in the plaster when investing, to secure the exact position of it in the vulcanite. It is claimed for this form of strengthener that no line or plane of weakness is determined in the vulcanite; that there can be no longitudinal slip on bending; that the plate may be finished and polished right down to the gold without possibility of stripping or peeling in wear; that the strengthener occupies the whole thickness of the plate, showing slightly on both surfaces, while affording a maximum of strength.—*Transactions of the Odontological Society of Great Britain.*

### INCREASE OF LEUCOCYTES IN THE BLOOD AFTER COLD BATHS.

Dr. W. S. Thayer publishes a paper on this subject in the *Johns Hopkins Hospital Bulletin* for April, based on some observations made by Dr. J. S. Billings, Jr., since last November. The latter gentleman found that after baths of twenty minutes' duration given at 70° F., blood taken from the lobe of the ear, in twenty cases of typhoid fever, showed that the average number of leucocytes before the bath was 7724 + and after the bath, 13,170 +. A further study was made of the relative proportion of the different varieties of the leucocytes to one another, and while there were constantly observed a slight diminution in the multinuclear neutrophiles and a slight increase in the uninuclear forms, there was no other difference in the proportion of varieties of leucocytes before and after the baths. The author is studying the questions of whether cases in which the bath is followed by an immediate reaction give the same results as those where there are cyanosis and coldness; of whether blood from parts that are cold and blue shows the same conditions as that taken from parts that are red and warm; of whether blood from a superficial cut with a lancet shows the same condition as that from a deep needle prick or possibly from a larger vein; of whether local applications of cold bring about the same result in a part as the general bath does; of whether there is any change in the number of red corpuscles in the cubic millimetre; and of how soon the increase in the number of leucocytes appears, and what its course is. Answers to these questions are necessary in order to determine whether this increase is general throughout the circulating blood, or whether it is only local and due to the accumulation of leucocytes in the smaller peripheral vessels. If the latter is the case, Winternitz's recent suggestion that the leucocytosis produced by cold baths exerts a destructive influence on any pathogenic organisms, and thus explains the increased "urotoxic coefficient" discovered by Roque and Weill to follow this treatment, is insufficient to explain the vaunted superiority of the cold-bath treatment. The scientific method displayed by the author in the study of this question is an example for other investigators, and we trust that he will pursue the investigation in order that he may determine the answers to the questions he has asked.—*New York Medical Journal.*

---

### IODOFORM *VERSUS* ARISTOL.

UNDER this head, Dr. Richard H. Gibbons, of Scranton, gives a very interesting account of his experience with aristol. The first case in which he employed it was after an operation for the removal of a cancerous mammary gland. The entire wound approximation was dusted with aristol. The lesion was dressed and closed for eight days, when it was found that a complete union had taken place. "Since then," says the author, "I have used aristol for all wound surfaces, exterior and cavital. In all operations about the anus and rectum I have found this remedy of great value."

Dr. Gibbons had equal success with aristol in diseased conditions of the eye, ear, nose, vagina, cervix, the female urethra, etc. He made satisfactory use of it also in suprapubic cystotomy and internal urethrotomy. The author adds that, "The powerful effects of aristol to promote rapid cicatrization" led him to employ it for special operations for the relief or cure of malignant disease of the female mammary gland. In the six cases cited the success achieved was remarkable. Concerning the value of aristol as a protective, Dr. Gibbons writes as follows: "The results which I have obtained in the use of aristol as a protection to wounds and ulcerated surfaces, and also as a stimulation to granulation, have been satisfactory to an extreme degree." Of its value in cœliotomy, he says, "In all cases of abdominal surgery I now use aristol, and find it to be the ideal protective, having had no cases of breaking down of the wound of entrance, as has happened in several cases where I have used iodoform."—*The Medical Bulletin*, March, 1893.

---

### THE VEGETABLE MERCURY OF BRAZIL.

In the April number of the *Annales de Dermatologie et de Syphiligraphie* there is an article by Dr. Cathelineau and Dr. Rebourgeon on this drug, founded on experiments in Professor Fournier's laboratory. It seems that in the equatorial regions of Brazil there grows a tree called by the natives *mururè.* It has not yet received its scientific name or been classified. By incisions into the bark of this tree a juice called vegetable mercury is obtained. In a work entitled *Formulario e guio medico*, published in Paris in 1884, Chernovitz stated that mururè juice was used in doses of a drachm, in half an ounce of water, the dose being repeated on every alternate day, according to the effects produced. It is an energetic purgative, and the natives use it especially in rheumatic affections, and above all in syphilis, whence its name. The bark is of a brick-red color. From its outer surface scales of a much deeper red are somewhat readily detached. Its inner surface is fibrous, grayish, and rather hard. The juice is a reddish liquid of rather a vinous odor and a sweetish taste. It is syrupy and of acid reaction. After being neutralized, it was administered to a rabbit, by intravenous injection, to the extent of four cubic centimetres to the kilogramme of the animal's weight, and caused death in thirty minutes. At the necropsy the stomach and intestine presented a vinous-red color. In the left ventricle of the heart there were reddish spots here and there. The kidneys were affected in like manner. In a dog an intravenous injection of four cubic centimetres to the kilogramme gave rise to the same phenomena, and produced death in forty-five minutes. Given by the mouth to the amount of eight cubic centimetres to the kilogramme, it caused death in twenty-four hours, and the lesions found were the same as have been mentioned.

Mururè juice is only partially soluble in distilled water, but the residue is soluble in alkalinized water. The authors experimented separately with the portion that is soluble in water and with that which dissolves only in alkalinized water. When the former was used, at the necropsy the heart and kidneys were found particularly affected, while the stomach and intestine presented merely a light coloration. When the latter was employed, death took place much more tardily, but the animals had intense diarrhœa, which was not observed in the others; moreover, at the post-mortem examination it was particularly the stomach and intestine that showed an intense red coloration, while there were no visible lesions of the heart and kidneys. The authors do not seem to have employed the drug remedially.—*New York Medical Journal*, June, 1893.

---

### INCREASED VITALITY HINDERS BACTERIA IN CARIES.

The history of teeth is not wholly determined by the circumstances of their formation. Many observations have proved that a slow circulation pervades the dentine, maintaining a correspondingly slow process of renewal and interstitial growth. This process certainly produces at times increased density, and the presumption almost amounts to certainty that it sometimes retrogrades and causes deterioration. The evidence of this is not easy to disentangle, but it grows in distinctness. It has long been observed that the teeth rapidly decay during or immediately after some attacks of prostrating sickness and some pregnancies. The cause usually assigned has been a pathological acidity of the buccal fluids; but it is now established by researches in bacteriology that, except at the very beginning, caries depends very little on external conditions; and therefore, if the long-recognized effects of sickness and pregnancy are facts, some cause must exist which acts within the tooth. And such a cause does exist, which is exactly in the line of our present study. Experiments made by the writer some years ago, but never published, showed that when decay occurs in fairly well-formed teeth, a zone of dentine immediately surrounding the cavity of decay has a higher specific gravity and yields more ash than a similar section from a sound side of the same tooth. This necessarily means a new deposit of lime-salts, apparently resulting from the irritation of decay. Now, such a deposit in itself is, of course, a local affair, but it implies a heightening of vital activity which must be traced back to nerve-centres. And it may well be assumed that, besides the increase of density, the increased vitality of the dentine hinders, *pro tanto*, the ravages of the bacteria. If, then, this be the normal check upon decay, anything which so cripples the nerve-centres as to prevent this opposition leaves the tooth defenceless to its enemies, and destruction is far more rapid. Now, to this failure of normal inhibition rather than to any local cause the quick decay accompanying disease and pregnancy must be ascribed.—J. Smith Dodge, M.D., D.D.S., in *Journal American Medical Association.*

---

### AN ALLEGED DEATH FROM EUCALYPTUS OIL.

An inquest was held at New Norfolk on Saturday, March 4, on the body of Victor Bradshaw, aged nine years and ten months, son of Mr. A. Bradshaw, of Back River. From the evidence it appeared that the deceased had been taking eucalyptus oil as a cure for cold, from which he and other members of the family were suffering. Finding that he was seriously ill, Dr. Neale was sent for, death ensuing shortly after the doctor's arrival. A post-mortem examination having been held, it was found that deceased must have taken one-half ounce of oil, the dose for an adult being five drops. The verdict of the jury was that death had been caused by an overdose of eucalyptus oil, with a rider that the word "poison" should be added to labels following the words "dose, one to five drops."—*The Australian Journal of Pharmacy.*

